# Supplementary figures and images for: Hydroxynonenal causes Langerhans cell degeneration in the pancreas of Japanese macaque monkeys
Source: PLoS One. 2021 Nov 8;16(11):e0245702. doi: 10.1371/journal.pone.0245702 (PMC8575276; doi:10.1371/journal.pone.0245702)

Fig. 7c

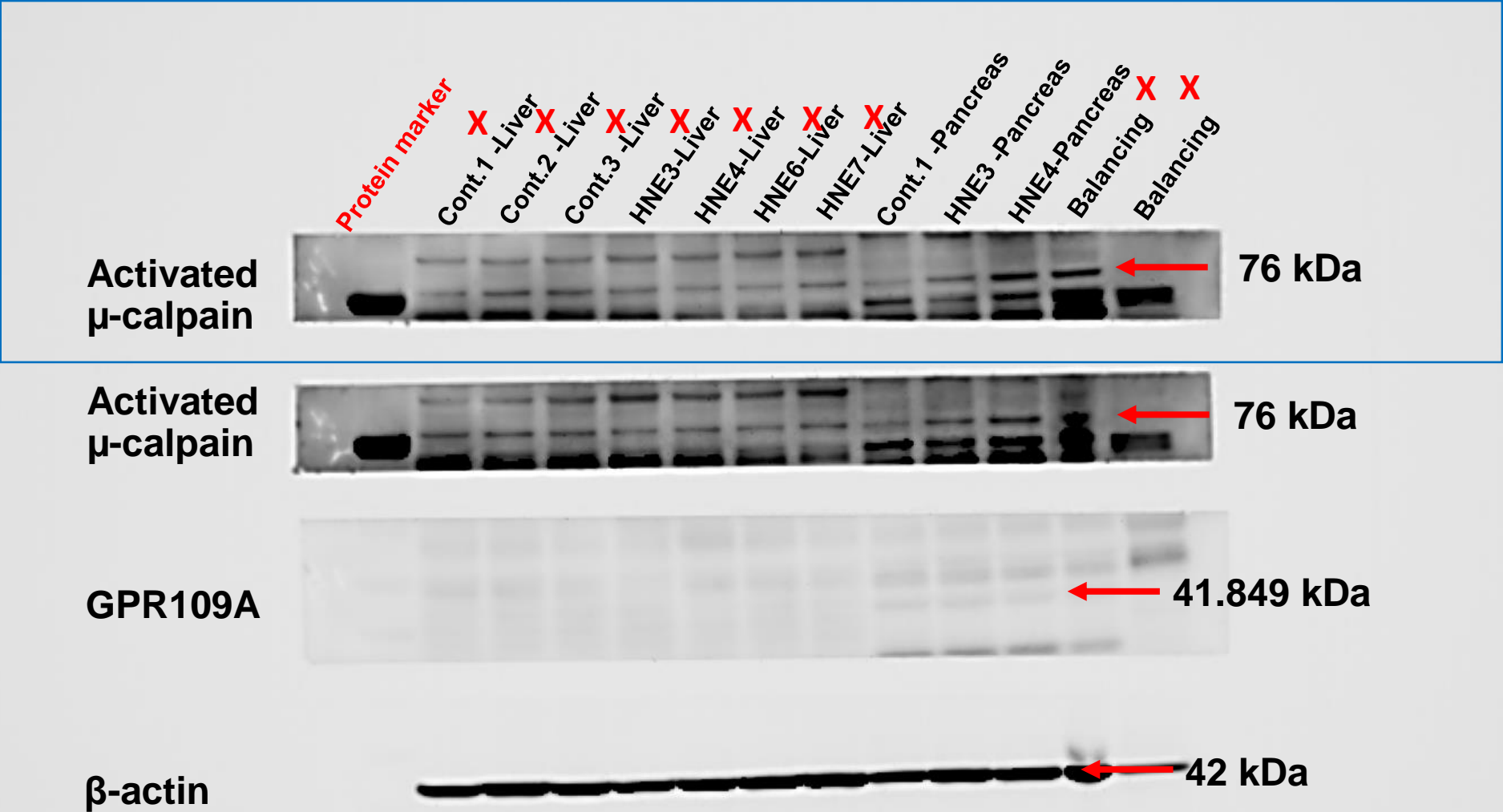

### Fig. 7c

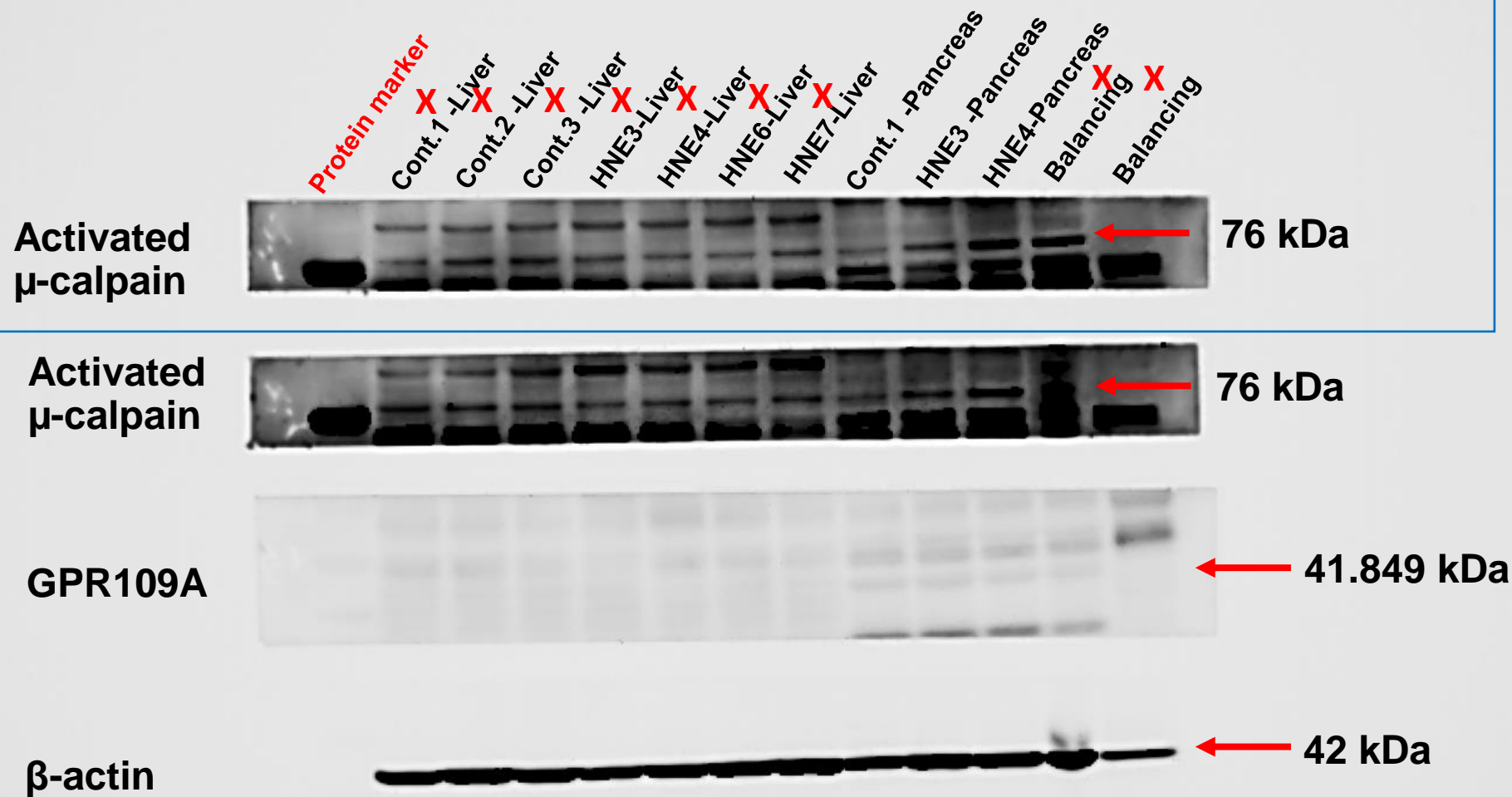

Fig. 7c

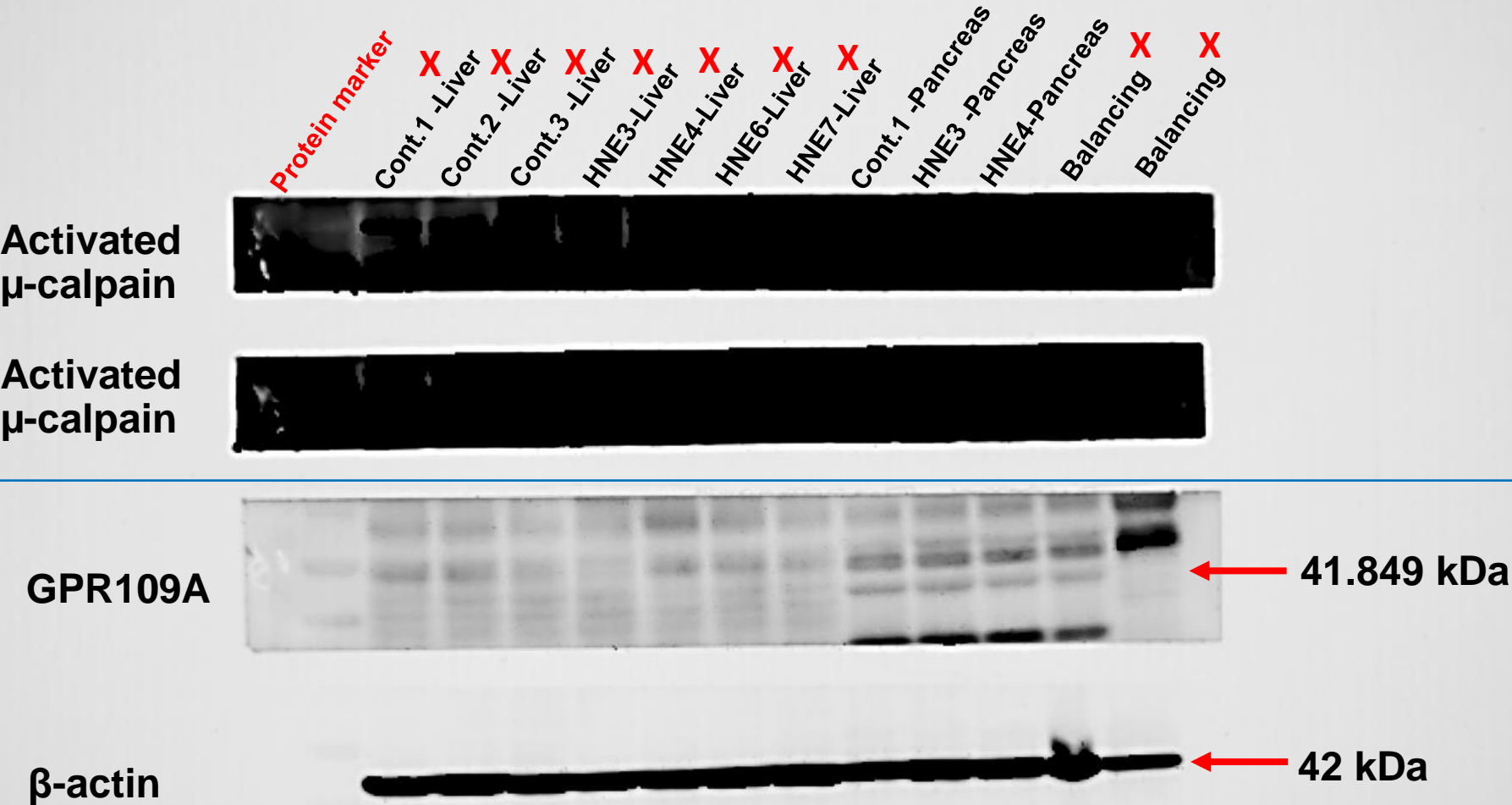

Fig. 7d

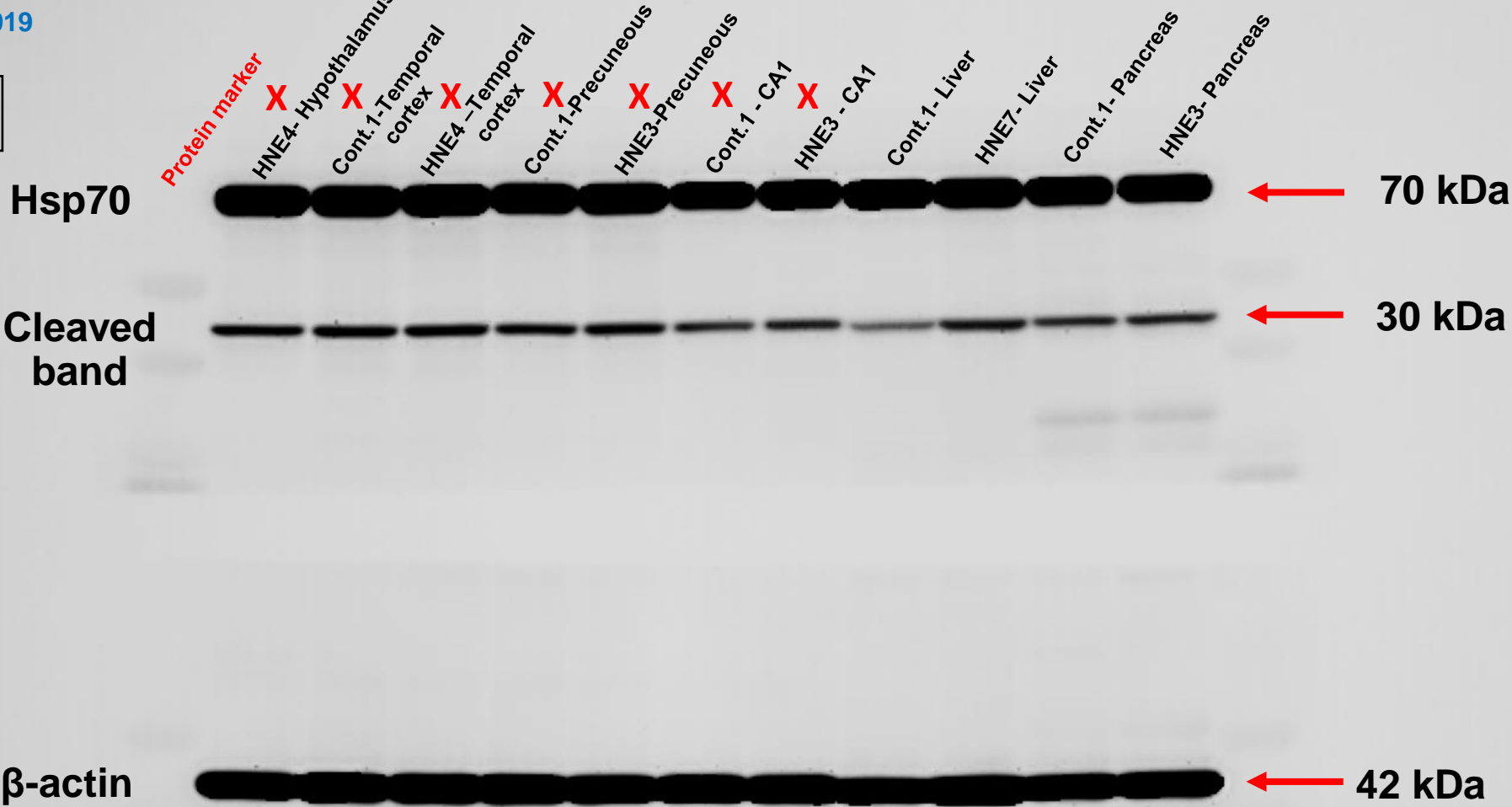

Supplement: S1 Data — (PDF) [file pone.0245702.s001.pdf]
